# Endexpiratory lung volume measurement correlates with the ventilation/perfusion mismatch in lung injured pigs

**DOI:** 10.1186/s12931-017-0585-y

**Published:** 2017-05-23

**Authors:** Jens Kamuf, Andreas Garcia-Bardon, Bastian Duenges, Tanghua Liu, Antje Jahn-Eimermacher, Florian Heid, Matthias David, Erik K. Hartmann

**Affiliations:** 1 Department of Anesthesiology, Medical Centre of the Johannes Gutenberg-University, Langenbeckstr. 1, 55131 Mainz, Germany; 2 Institute of Medical Biostatistics, Epidemiology and Informatics, Medical Centre of the Johannes Gutenberg-University, Mainz, Germany

**Keywords:** ARDS, Endexpiratory lung volume, Ventilation/perfusion mismatch, Pig model

## Abstract

**Background:**

In acute respiratory respiratory distress syndrome (ARDS) a sustained mismatch of alveolar ventilation and perfusion (V_A_/Q) impairs the pulmonary gas exchange. Measurement of endexpiratory lung volume (EELV) by multiple breath-nitrogen washout/washin is a non-invasive, bedside technology to assess pulmonary function in mechanically ventilated patients. The present study examines the association between EELV changes and V_A_/Q distribution and the possibility to predict V_A_/Q normalization by means of EELV in a porcine model.

**Methods:**

After approval of the state and institutional animal care committee 12 anesthetized pigs were randomized to ARDS either by bronchoalveolar lavage (*n* = 6) or oleic acid injection (*n* = 6). EELV, V_A_/Q ratios by multiple inert gas elimination and ventilation distribution by electrical impedance tomography were assessed at healthy state and at five different positive endexpiratory pressure (PEEP) steps in ARDS (0, 20, 15, 10, 5 cmH_2_O; each maintained for 30 min).

**Results:**

V_A_/Q, EELV and tidal volume distribution all displayed the PEEP-induced recruitment in ARDS. We found a close correlation between V_A_/Q < 0.1 (representing shunt and low V_A_/Q units) and changes in EELV (spearman correlation coefficient −0.79). Logistic regression reveals the potential to predict V_A_/Q normalization (V_A_/Q < 0.1 less than 5%) from changes in EELV with an area under the curve of 0.89 with a 95%-CI of 0.81–0.96 in the receiver operating characteristic. Different lung injury models and recruitment characteristics did not influence these findings.

**Conclusion:**

In a porcine ARDS model EELV measurement depicts PEEP-induced lung recruitment and is strongly associated with normalization of the V_A_/Q distribution in a model-independent fashion. Determination of EELV could be an intriguing addition in the context of lung protection strategies.

## Background

Within the lung adequate matching of alveolar ventilation and perfusion determines sufficient gas exchange. In healthy subjects the ventilation/perfusion ratio (V_A_/Q) is widely normally distributed with small physiological shunt and low V_A_/Q fractions. Development of an acute respiratory distress syndrome (ARDS) causes high amounts of atelectatic or poorly ventilated lung areas going along with an altered perfusion [1, 2], which leads to a sustained V_A_/Q mismatch and consecutive gas exchange impairment. V_A_/Q ratios can hardly be measured in the clinical routine, and can only be estimated from conventional blood gas analysis or shunt calculation. Sophisticated imaging technologies like single photon emission tomography or positron emission tomography lack feasibility in critically ill patients [3]. The multiple inert gas elimination technique (MIGET) is regarded as experimental gold standard for V_A_/Q measurement, though also has feasibility concerns despite advances in technology, automatization and time requirements [4]. Another novel, non-invasive technique for evaluation of lung aeration without radiation exposition is the assessment of the functional residual capacity or, if a positive end-expiratory pressure (PEEP) is applied, the end-expiratory lung volume (EELV) based on multiple breath nitrogen washout/washin developed by Olegard et al. [5]. This technique provides a reliable EELV measurement in vivo and correlates well to computer tomography-based volumetric assessment (*r* ^2^ = 0.89) [6].

EELV measurement does not distinguish between distinct V_A_/Q fractions and addresses primarily the ventilatory pattern, but not alterations of pulmonary perfusion. Hence, it is not known, if EELV measurement can predict V_A_/Q normalization. Hence, we sought to further analyze the association between EELV and V_A_/Q ratio in porcine ARDS models. We hypothesized that (1) EELV changes in different lung injury models reflect PEEP-induced recruitment and (2) EELV measurement indirectly predicts normalization of V_A_/Q.

## Methods

After approval of the institutional and state animal care committee (Landesuntersuchungsamt Rheinland-Pfalz, Koblenz, Germany; approval number G12-1-059) we performed this prospective randomized animal study in accordance with the international guidelines for the care and use of laboratory animals. This manuscript adheres to the applicable EQUATOR guidelines.

### Anesthesia and instrumentation

Twelve healthy male pigs (sus scrofa domestica, weight: 26–33 kg) were sedated (ketamine 4 mg kg^−1^, azaperon 8 mg kg^−1^ intramuscular) and delivered by a local breeder. After establishing an intravenous line anesthesia was induced and maintained by propofol (8–12 mg kg^−1^ h^−1^) and fentanyl (0.1–0.2 mg kg^−1^ h^−1^). A single dose atracurium (0.5 mg kg^−1^) was administered to facilitate orotracheal intubation. Ventilation (Respirator: Engström Carestation®, GE Healthcare, Germany) was started in pressure-controlled mode with a tidal volume (V_t_) of 7 ml kg^−1^, PEEP of 5 cmH_2_O, fraction of inspired oxygen (FiO_2_) of 0.4 and a variable respiratory rate to maintain normocapnia. A balanced electrolyte solution (Sterofundin iso, B. Braun, Germany) was continuously infused at a rate of 5 ml kg^−1^ h^−1^. Vascular catheters were placed ultrasound-guided: an arterial line, a pulse contour cardiac output catheter (PiCCO, Pulsion Medical Systems, Germany), a central venous line and a 7.5-French introducer for a pulmonary arterial catheter were inserted via femoral vascular access. Esophageal pressure was measured by a balloon-catheter to enable transpulmonary pressure calculation. Respiratory and extended hemodynamic parameters were recorded continuously (Datex S/5, GE Healthcare, Germany). Further respiratory parameters and measurements were recorded by the respirator. Normothermia was maintained by body surface warming.

### Extended respiratory monitoring

The EELV was determined semi-automatically through the Engström Carestation by means of the nitrogen washout/washin method with a FiO_2_ change of 0.1 as described by Olegard and co-workers [5]. All measurements were done twice and the mean value was used. All EELV measurements were referenced to the animals’ individual baseline value in a healthy state (EELV/EELV_Baseline_ [%]). The V_A_/Q-distribution was determined using micropore membrane inlet mass spectrometry-MIGET (MMIMS-MIGET, Oscillogy LLC, Folsom, PA, USA). After steady-state infusion of a saline solution containing six inert gases (sulphur hexafluoride, krypton, desfluran, enfluran diethyl ether and acetone) in subclinical and non-toxic dosage for 30 min, synchronous mixed-venous and arterial blood samples were taken. Following ARDS induction the saline solution was infused continuously to guarantee a steady state of inert gas retention and elimination. Sampling procedure and data processing were carried out according to previously reported protocols [7]. Hypoventilated compartments (V_A_/Q < 0.1) representing the sum of true shunt (V_A_/Q < 0.05) and low V_A_/Q areas, normal and hyperventilated compartments (high V_A_/Q) are reported as fraction of cardiac output [7, 8]. To analyze the regional ventilation distribution we used an electrical impedance tomography device (EIT; Goe-MF II, CareFusion, San Diego, CA, USA) that records thoracic bioimpedance variations associated with tidal ventilation. The electrodes were placed on a transverse lung section just below the axilla. The regional ventilation distribution was examined for the non-dependent, central, and dependent lung regions (Levels L1-L3) as percentage of the global tidal amplitude [9].

### Study protocol

Following instrumentation, we set the F_i_O_2_ to 1.0 and conducted a lung recruitment maneuver (plateau pressure 40 cmH_2_O for 10 s). Then baseline parameters were assessed at healthy state. Afterwards the pigs were randomized to ARDS induction by either lung lavage (LAV, *n* = 6) followed by 1 h of high tidal volume ventilation [7] or by central venous oleic acid injection (OAI, *n* = 6) to depict different etiologies and kinetics of ARDS. For LAV the endotracheal tube was clamped in inspiration. Then 30 ml kg^−1^ of warmed balanced saline solution were instilled and directly drained by gravity. Lavage procedures were repeated within 10 min intervals until a PaO_2_/FiO_2_ ratio ≤ 250 mmHg was achieved. Afterwards animals were ventilated for 1 h in pressure-controlled mode with the following settings: V_t_ of 15 ml kg^−1^, PEEP 0, FiO_2_ 1.0, and respiratory rate to maintain normocapnia. After this hour V_t_ was reduced to 7 ml kg^−1^. For OAI 0.1 ml kg^−1^ of oleic acid (cis-9-octadecenoic acid) were solved in 20 ml saline solution and injected via the central venous line in fractions of 2 ml every 3 min. The procedure was repeated with another 0.1 ml kg^−1^ after 15 min, if the PaO_2_/FiO_2_ was higher than 200 mmHg. After ARDS criteria were fulfilled respirator settings were adapted: V_t_ 7 ml kg^−1^, FiO_2_ 1.0. We used five different PEEP steps throughout the experiment (0, 20, 15, 10, 5 [cmH_2_O]), whereby each step was maintained for 30 min to achieve stable conditions. If necessary to warrant stable hemodynamics during the experiments (mean arterial pressure > 60 mmHg), norepinephrine was administered. After finishing all measurements, the animals were killed in deep general anesthesia by injection of 200 mg propofol and 40 mmol potassium.

### Statistics

Physiological parameters are displayed as mean and standard deviations. A repeated measurement ANOVA was performed to compare the parameters between the different PEEP levels using the F-test for least squares means and Bonferroni-adjusted post-hoc t-tests for the pairwise comparisons. To investigate the influence of PEEP and injury model on the relative change in EELV from baseline, PaO_2_ and tidal volume distribution per level linear mixed models were fitted with PEEP level and lung injury model as fixed factors and a random intercept to account for intra-subject correlations. For PaO_2_ an interaction term PEEP *injury model was included. F-tests and t-tests for least squares means (LS-means) differences were calculated for the fixed effects and pairwise comparisons, respectively. In the interaction model for PaO_2_ the pairwise comparisons between PEEP steps were derived from the average LS-means over the two injury models and the comparison between the two injury models was derived from the average LS-means over the PEEP steps. The changes in V_A_/Q between the different PEEP levels and baseline are evaluated by Wilcoxon signed rank tests. Correlation between the relative change in EELV and V_A_/Q is described by a scatter plot and the spearman rank correlation coefficient. Further associations between EELV respectively V_A_/Q < 0.1 and PaO_2_, lung compliance, and airway driving pressure were analyzed. The discriminating ability of relative change in EELV to distinguish between V_A_/Q fractions < 0.1 above or below 5, 10, 15 and 30% is described by receiver operating characteristic (ROC) curves as derived from logistic regression. As this is an explorative study no adjustments for multiple testing have been done. *P*-values are given for descriptive reasons only and must be interpreted with caution due to the large number of tests.

## Results

A total of 12 animal experiments were included in this study. One animal didn’t tolerate the zero PEEP setting after induction of ARDS, so we applied a PEEP of 5 cmH_2_O for the initial step. Another animal died following the last drop to PEEP 5 cmH_2_O in ARDS. Lung injury induction led to sustained gas exchange impairment, increase of the extravascular lung water content and pulmonary arterial hypertension. The physiological parameters are summarized in Table 1.

**Table 1:** Physiological parameters

	Baseline	P0	P20	P15	P10	P5
MAP [mmHg]	82 ± 8	81 ± 17	69 ± 12^a, b^	76 ± 13	75 ± 12	74 ± 12
CVP [mmHg]	7 ± 1	6 ± 2	9 ± 2	9 ± 2	8 ± 2	6 ± 2
MPAP [mmHg]	17 ± 2	35 ± 5^a^	32 ± 5^a^	29 ± 6^a^	30 ± 9^a^	28 ± 6^a^
CO [l min^−1^]	3.8 ± 0.6^b, c^	5.2 ± 1.6	3.7 ± 0.9^b, c^	3.8 ± 0.8^b, c^	4.2 ± 1.0^c^	5.6 ± 1.5
EVLWI [ml kg^−1^]	13 ± 4	25 ± 10^a^	22 ± 9^a^	22 ± 9^a^	23 ± 9^a^	24 ± 10^a^
V_t_ [ml kg^−1^]	6.6 ± 0.4	6.9 ± 0.4	6.9 ± 0.5	6.9 ± 0.4	6.9 ± 0.4	7.0 ± 0.4
RR [min^−1^]	36 ± 4	35 ± 3	44 ± 3^a, b^	44 ± 3^a, b^	43 ± 3^a, b^	41 ± 4^a, b^
etCO_2_ [mmHg]	39 ± 2	40 ± 6^c^	46 ± 4^a, b, d, c^	42 ± 3^d, c^	38 ± 2	36 ± 3
P_peak_ [cmH_2_O]	15 ± 2	29 ± 8^a^	40 ± 3^a, b, e, d, c^	32 ± 4^a^	29 ± 6^a^	28 ± 7^a^
PEEP [cmH_2_O]	5 ± 1	1 ± 1^a, e, d, c^	21 ± 1^a, b, e, d, c^	16 ± 1^a, d, c^	10 ± 1^a, c^	5 ± 1
P_TP_ [cmH_2_O]	5 ± 3	18 ± 6^a^	25 ± 3^a, b, e, d, c^	19 ± 4^a^	17 ± 6^a^	17 ± 9^a^
∆P [cmH_2_O]	8 ± 2	25 ± 5^a, e, c^	18 ± 3^a, b^	15 ± 4^a^	18 ± 7^a^	22 ± 7^a, e, d^
C_dyn_ [ml cmH_2_O^−1^]	22 ± 7	8 ± 2^a^	12 ± 3^a, b^	14 ± 4^a, b, c^	13 ± 5^a, b^	10 ± 3^a^

*MAP* mean arterial pressure, *CVP* central venous pressure, *MPAP* mean pulmonal arterial pressure, *CO* cardiac output, *EVLWI* extravascular lung water index, *V*_*t*_ tidaL volume, *RR* respiratory rate, *etCO*_*2*_ endtidal carbon dioxide, *P*_*peak*_ peak inspiratory pressure, *PEEP* positive endexpiratory pressure, *P*_*TP*_ transpulmonary pressure, ∆*P* dynamically calculated airway driving pressure (P_peak_-PEEP), *C*_*dyn*_ dynamic lung compliance

*P* < 0.05

^a^ vs. BLH

^b^ vs. P0

^c^ vs. P5

^d^ vs. P10

^e^ vs P15

**Fig. 1:**
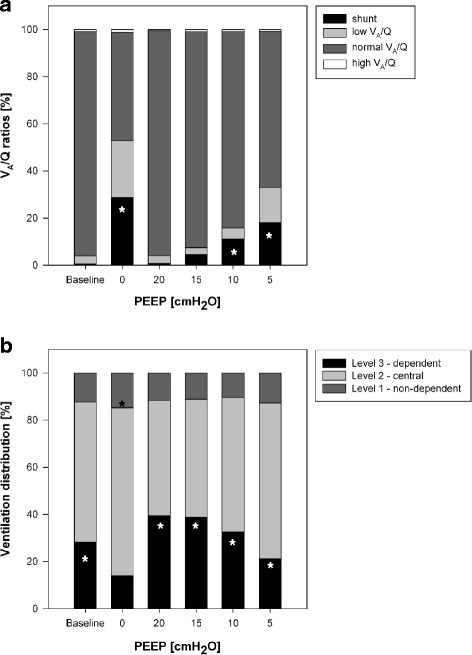
Influence of PEEP-induced lung recruitment on V_A_/Q distribution and ventilation distribution. The V_A_/Q distribution was measured by multiple inert gas elimination (*upper* graph) and the ventilation distribution by electrical impedance tomography (*lower* graph). Key statistical findings (*p* < 0.05) are marked by *

**Fig. 2:**
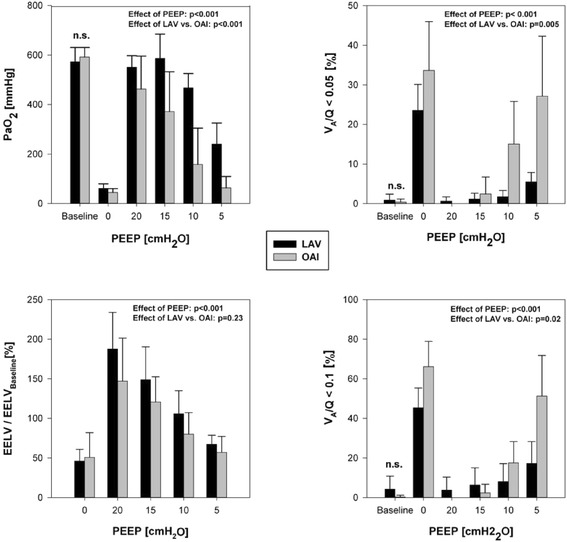
Model-dependent effect of PEEP-induced lung recruitment in bronchoalveolar lavage (LAV)-induced versus oleic acid injection (OAI)-ARDS. Impact on PaO_2_ and EELV (*upper/lower left*) and V_A_/Q fractions < 0.05 (*shunt; upper right*) and < 0.1 (all hypoventilated areas; *lower right*). n.s. non-significant

Figure 1 shows the PEEP-related changes of V_A_/Q distribution (upper graph) and regional tidal ventilation (lower graph): shunt values were significantly higher at PEEP 0 (*p* < 0.001), PEEP 10 (*p* < 0.05) and PEEP 5 (*p* < 0.05) compared to baseline. Low V_A_/Q was significantly higher at PEEP 0 (*p* < 0.05) compared to baseline, at PEEP 5 significance was marginally missed (*p* = 0.058). No relevant high V_A_/Q ratios developed despite the PEEP changes. The regional ventilation distribution shows a PEEP dependency in ARDS with reduced tidal ventilation in the dependent lung areas and a corresponding increase in the central and non-dependent regions. The non-dependent region exhibits decent, but significant (each *p* < 0.05) changes in tidal ventilation between PEEP 0 and all other PEEP levels. Tidal ventilation in the dependent lung area also responds to the PEEP-induced recruitment with significant difference between all PEEP steps except PEEP 15 versus PEEP 20 (*p* = 0.76). Induction of ARDS significantly reduced the EELV in both groups (Fig. 2). Considerable alterations occurred in response to the consecutive PEEP escalation (*p* < 0.001) without difference between the two lung injury models (*p* = 0.19). The PaO_2_ significantly changed with each PEEP step as compared to baseline (*p* < 0.001). Furthermore, there revealed to be a model-dependent influence in the resulting PaO_2_ (*p* < 0.001).

**Fig. 3:**
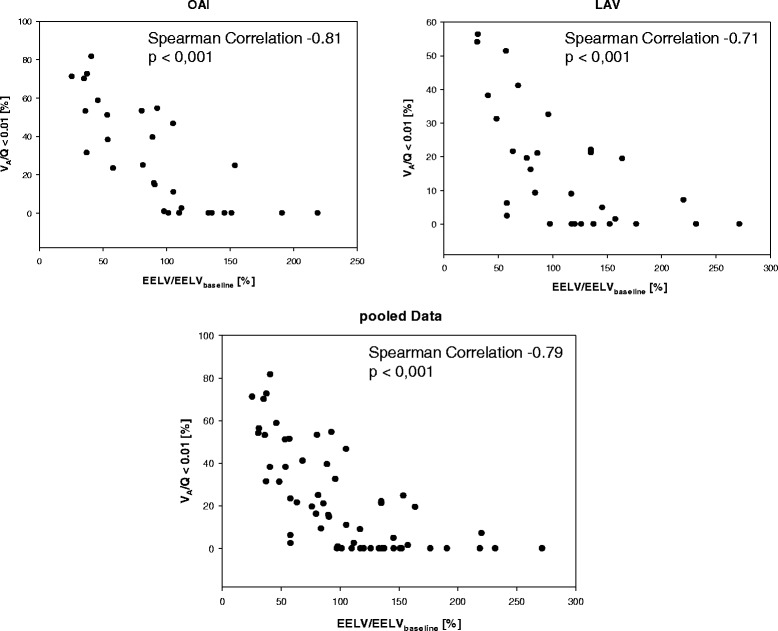
Correlation between V_A_/Q <0.1 and EELV. Separate correlations for oleic-acid injection (OAI)-ARDS (*upper left*), bronchoalveolar lavage (LAV)-ARDS (*upper right*) and the pooled data *(lower graph*)

**Fig. 4:**
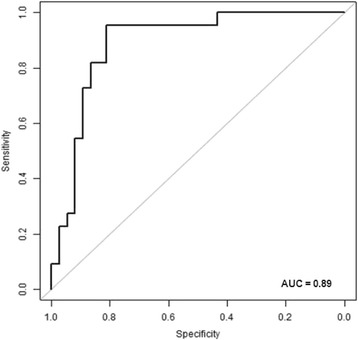
Receiver operating characteristic curve for the prediction of V_A_/Q normalization by EELV measurements. AUC: area under the curve

Despite differences in response to PEEP-induced recruitment the EELV is highly correlated to V_A_/Q ratios < 0.1 with a spearman rank correlation coefficient of −0.79 (Fig. 3). The separate coefficients for the two ARDS models were −0.71 (LAV) and −0.81 (OAI). Logistic regression showed a ROC curve with an area under the curve (AUC) of 0.89 with a 95%-CI of [0.81; 0.96] (Fig. 4) when discriminating between V_A_/Q fractions < 0.1 above or below 5%. A Youden index of 0.77 is observed when applying a threshold of 96.8% for the relative change in EELV from baseline. The same threshold applied to discriminate between V_A_/Q fractions < 0.1 above or below 10 and 15% results in a Youden index of 0.73, respectively. For V_A_/Q fractions < 0.1 below 30% the best Youden index of 0.7 is observed when defining a threshold for the EELV-change of 57.5%. Additional analyses revealed that changes in EELV are associated with changes in PaO_2_(Spearman correlation coefficient 0.74, *p* < 0.001), in lung compliance (Spearman correlation coefficient 0.48, *p* < 0.001) and dynamically calculated driving pressure (peak pressure – PEEP; Spearman correlation coefficient −0.46, *p* < 0.001). V_A_/Q ratios < 0.1 are also correlated with lung compliance (Spearman correlation coefficient −0.65, *p* < 0.001) and airway driving pressure (Spearman correlation coefficient 0.66, *p* < 0.001).

## Discussion

The present study examines the association EELV and V_A_/Q in lung injured pigs: a close correlation (*R* = −0.79) and a ROC with an AUC of 0.89 were found. The ROC curve is a tool to evaluate the predictive potency of a parameter by examining sensitivity and specificity, whereby the AUC is regarded a quality marker [10]. Regarding this, our data show the possibility to predict changes in V_A_/Q ratios < 0.1 with high accuracy by measuring EELV in pigs. Our results retrieve a strong association, that even in ARDS if the EELV is close to the individuals’ baseline, V_A_/Q fractions < 0.1 are smaller than 5%. The ability of EELV to discriminate V_A_/Q fractions < 0.1 smaller than 10 or 15% showed similar results. Additional, but less considerable associations were found between EELV and lung compliance respectively dynamic driving pressure.

Measurement of EELV by means of nitrogen washout/washin is a novel technique that has been made available for bedside use due to integration in intensive care respirators. The technique was successfully validated against computer tomography and helium dilution technique [6]. EELV measurements show a good correlation to PEEP-induced lung volume changes, if leakage-related artefacts are prevented (*r* ^2^ = 0.80) [11]. The ratio of PEEP-induced changes in EELV and the baseline functional residual capacity can be used to differentiate patients with a high or low recruitment potential [12]. In pigs, EELV significantly correlates but underestimates the computer tomography-based volumetry with an AUC of at least 0.73 for EELV changes larger than 150 ml [13]. ARDS induces a shift towards hypoventilated areas (low V_A_/Q) and shunt [7]. The MIGET-derived V_A_/Q is able to depict ventilation [4, 14] as well as perfusion impairment [8, 15]. Our data reveal that during PEEP-induced recruitment measurement of EELV reliably predicts the occurring V_A_/Q normalization. Theoretically, derecruitment and atelectasis formation subsequently leading to EELV decrease is inherent to V_A_/Q mismatch and shunt increase. This simplified model, however, neither considers poorly ventilated but non-atelectatic lung tissue nor altered perfusion that is capable to affect V_A_/Q independently of lung recruitment. Though, the hemodynamic response to PEEP escalation in our data does not suggest the latter. Considerable low V_A_/Q fractions occur not only in our data (Fig. 1) but also in patients suffering from ARDS [16] and substantially influence V_A_/Q mismatch. Conversion of low to normal V_A_/Q areas may not be fully reflected by EELV increase, which explains that PaO_2_ can increase despite constant EELV [17]. This highlights the need for differentiation between mere anatomical and functional recruitment [18]. To our knowledge, the present study is the first to approve a direct predictive value of EELV on V_A_/Q despite this complex underlying pathophysiology. Several factors that alter pulmonary perfusion like hypovolemia or impaired hypoxic pulmonary vasoconstriction (HPV) may also influence the reported correlation. In lipopolysaccharide-induced sepsis HPV is blunted [19], which contributes significantly to V_A_/Q mismatch and may interfere with the V_A_/Q-EELV association. Pigs, in this context, exhibit a high degree of HPV [20], which is not compromised by LAV-induced ARDS, but may be reduced by OAI to a certain degree [21]. Accordingly, the predictive value of EELV needs to be interpreted in the individual context.

Our results further explain the findings of Krause et al. [22]: they showed an optimal effect of inhaled nitric oxide in lung injured pigs when EELV was similar to the pre-injury state. This is associated with an intact V_A_/Q distribution and may provide ideal conditions for drug inhalation. The declining effect with further increase in PEEP and EELV on the other hand should be related to a shift from normal to high V_A_/Q areas and impaired perfusion. The complex combination of several pathomechanisms in ARDS can hardly by mimicked in full extent by experimental models [23]. Assessment of lung recruitability is crucial in ARDS patients: the individual recruitability may influence the ventilatory management, PEEP requirement, and even survival [24]. In experimental research the response to ventilatory interventions may very well depend on the chosen model and its underlying pathomechanism [9]. To reduce the probability of a mere model-dependent effect, we chose to examine two common but different models of ARDS with LAV and OAI, which represent surfactant depletion-related alveolar collapse and atelectasis respectively capillary leakage-induced edema and alveolar flooding [25]. Based on these considerations LAV-injured lungs respond well to PEEP or recruitment maneuvers [2]. The OAI model, however, is characterized by a need for higher PEEP to improve oxygenation [26]. Low V_A_/Q areas are considerably larger in OAI and less recruitable [2]. Furthermore, an only moderate correlation of PEEP-induced EELV or with PaO_2_ (*r* ^2^ = 0.53) was found in a previous study [27], which may be caused by delayed recruitment from low to normal V_A_/Q ratios that contribute to the gas exchange to a lesser degree than directly recruited atelectasis. Consistent with this data, we found a significantly higher PaO_2_ and decrease of shunt and low V_A_/Q ratios at lower PEEP in lung LAV-injured pigs, but no model-dependent differences in EELV. This may be caused higher variances and the smaller EELV changes between the PEEP steps as assumed by Richard et al. [13]. In both models, though, EELV and V_A_/Q < 0.1 are separately correlated despite different response in the PEEP trial. Hence given a positive PEEP response, EELV referenced to its individual baseline reliably predicts V_A_/Q normalization independent from the model related recruitment characteristics.

The present study has some limitations. PEEP-induced EELV normalization is not necessarily lung protective by itself. A forced PEEP raise may lead to injurious hyperinflation of the persisting healthy lung tissue even without achieving a fully recruited lung [28]. In our model sustained hyperinflation is unlikely due to only minimal occurrence of high V_A_/Q ratios going along with decent changes of tidal ventilation in the non-dependent lung areas. Nevertheless, we cannot fully rule out overdistension, but this was not the main focus of this study and may be addressed in further examinations. Increased endtidal CO_2_ levels in the high PEEP settings may point towards increasing dead space to which we cannot provide sufficient physiological explanations from the present data. Recent data highlight the role of the driving pressure (plateau pressure – PEEP) to determine the meaningfulness of respiratory modifications [29]. Instead, the present protocol only allowed for calculation of the dynamic, but yet less validated, variant of airway driving pressure (peak pressure – PEEP) [30]. If below harmful thresholds, patients might benefit from V_A_/Q optimization without the risk of sustained further injury. Hence, EELV measurement can represent only one cornerstone to optimize lung recruitment and gas exchange in a lung protective ventilation strategy, if severe hyperinflation is prevented by additional monitoring methods [31]. We found a good discriminating ability for V_A_/Q fractions < 0.1 below 5, 10 and 15 when applying a threshold of 96.8% of baseline EELV and a slightly minor discriminating ability for V_A_/Q fractions < 0.1 below 30% when applying a threshold of 57.5% for the EELV change. 96.8% of the baseline EELV revealed to be the optimum cut-off. But the individual amounts of V_A_/Q < 0.1, which is just tolerable to ensure sufficient oxygenation in ARDS patients, needs to be confirmed in clinical studies. We referenced the EELV to the individual baseline value rather than comparing the absolute EELV. This concept has been previously assumed [22] and prevents variances due to different individual conditions. Dellamonica et al. developed a method to determine this baseline even in ARDS patients by using a prolonged exhalation without direct measurements at zero PEEP or derecruitment [12].

## Conclusion

In porcine ARDS models measurement of EELV by nitrogen washout/washin retraces PEEP-induced recruitment, V_A_/Q normalization and ventilation distribution. The EELV is strongly associated with the extent of V_A_/Q ratios < 0.1. An EELV close to the individual baseline value predicts normalization of the V_A_/Q with a high sensitivity.

Automatized nitrogen washout/washin is an interesting bedside tool for EELV measurement and even indirect assessment of the V_A_/Q mismatch in ARDS. Implementation of EELV in lung protection strategies could facilitate the decision, if patients benefit from further recruitment.

## Abbreviations

ANOVA: Analysis of variance
ARDS: Acute respiratory distress syndrome
AUC: Area under the Curve
EELV: Endexpiratory lung volume
EIT: Electrical impedance tomography
etCO_2_: endtidal CO_2_
FiO_2_: Fraction of inspired oxygen
HPV: Hypoxic pulmonary vasoconstriction
LAV: ARDS induced by bronchoalveolar lavage
LS: Least squares
MAP: Mean arterial pressure
MMIMS-MIGET: Micropore membrane inlet mass spectrometry - multiple inert gas elimination technique
OAI: ARDS induced by central-venous injection of oleic acid
PaO_2_: Arterial partial pressure of oxygen
PEEP: Positive end-expiratory pressure
ROC: Receiver operating characteristic
V_A_/Q: Ventilation/perfusion distribution
V_t_: Tidal volume
